# Antioxidant and Health-Related Effects of Tannins: From Agri-Food By-Products to Human and Animal Health

**DOI:** 10.3390/antiox15010104

**Published:** 2026-01-13

**Authors:** Luca Camarda, Roberta Budriesi, Ivan Corazza, Maria Frosini, Carla Marzetti, Laura Beatrice Mattioli

**Affiliations:** 1 Department of Pharmacy and Biotechnology (FaBiT), Food Chemistry and Nutraceutical Laboratory, Alma Mater Studiorum-University of Bologna, Via Belmeloro 6, 40126 Bologna, Italy; laurabeatrice.mattioli@unibo.it; 2 Health Sciences and Technologies-Interdepartmental Center for Industrial Research (CIRI-SDV), Alma Mater Studiorum-University of Bologna, Via Massarenti 9, 40126 Bologna, Italy; ivan.corazza@unibo.it; 3 Department of Medical and Surgical Sciences (DIMEC), Alma Mater Studiorum-University of Bologna, 40138 Bologna, Italy; 4 Department of Life Sciences, University of Siena, Via A. Moro 2, 53100 Siena, Italy; maria.frosini@unisi.it; 5 Valsambro S.r.l., Via Cairoli 2, 40121 Bologna, Italy; carla.marzetti@valsambro.it

**Keywords:** antioxidant activity, tannins, condensed tannins, hydrolysable tannins, agri-food by-products, redox modulation, oxidative stress, nutraceutical antioxidants, gut–brain axis, One Health

## Abstract

**Background:** Agri-food by-products are increasingly recognized as valuable sources of tannins, whose antioxidant properties represent the primary driver of their biological activity across human and animal health. The strong redox-modulating capacity of condensed and hydrolysable tannins provides a unifying mechanistic explanation for their effects on inflammation, metabolism, gut integrity and neuroprotection. **Methods:** This narrative review synthesizes evidence obtained through a structured literature search across major databases, selecting studies that investigated antioxidant mechanisms of tannin-rich matrices from plant- and processing-derived residues. **Results:** Condensed tannins, particularly proanthocyanidins, consistently display potent antioxidant activity through radical scavenging, metal chelation and activation of endogenous defenses, thereby underpinning their anti-inflammatory, anti-ischemic, neuroprotective and metabolic actions. Hydrolysable tannins similarly exert strong antioxidative effects that support antimicrobial activity, enzyme modulation and protection against neuroinflammation. In animals, the antioxidant capacity of tannins translates into improved oxidative balance, enhanced immune status, reduced tissue damage, better feed efficiency and mitigation of oxidative stress-linked methane emission pathways. **Conclusions:** Antioxidant activity emerges as the central, cross-species mechanism through which tannins mediate diverse health benefits. Tannin-rich agri-food by-products therefore represent promising sustainable antioxidant resources, although their efficacy remains influenced by tannin class, degree of polymerization and dosage, warranting further mechanistic and translational research.

## 1. Introduction

On a dry matter basis, Agri-Food Waste and By-Products (AFWBs) are composed of considerable amounts of carbon (51.2%), hydrogen (7.2%), oxygen (38.1%), nitrogen (2.8%), and sulfur (0.7%), whose safe management is necessary to avoid adverse environmental effects, including soil and water contamination as well as increased greenhouse gas emissions [1]. Globally, AFWBs amount to approximately 1.3 billion tons each year, resulting from both primary and secondary processes along the supply chain. These losses originate from various stages, including production and postharvest handling—which account for about 75% of total food losses, as observed in developing African countries—as well as from consumer-level wastage, particularly common in industrialized regions such as North America and Europe [2]. The agri-food industry, in particular, contributes significantly to this issue, generating large quantities of organic residues (biomass) that can reach up to 140 billion tons annually, although a substantial portion of these materials does not directly derive from food waste [3].

In recent years, the One Health concept has gained global relevance as a comprehensive and integrative framework that recognizes the intricate interdependence between human, animal, and environmental health [4]. This multidisciplinary approach emphasizes that sustainable well-being cannot be achieved by addressing these domains in isolation, but rather through coordinated actions that preserve ecosystem integrity, promote animal welfare, and ensure food and nutritional security [5]. Within this perspective, the transition toward circular and sustainable food systems plays a pivotal role, fostering the responsible use of natural resources and the recovery of valuable compounds from agricultural and food processing chains.

Oxidative stress arises from an imbalance between the production of reactive oxygen species (ROS) and the capacity of endogenous antioxidant defenses to neutralize them. Excessive ROS accumulation contributes to cellular damage by oxidizing lipids, proteins, and nucleic acids, thereby playing a central role in the onset and progression of chronic and degenerative diseases, including cardiovascular, metabolic, inflammatory, and neurodegenerative disorders. Antioxidant compounds can counteract these processes by scavenging free radicals, chelating pro-oxidant metals, and modulating redox-sensitive signaling pathways, ultimately contributing to the maintenance of cellular homeostasis and human health [6,7,8].

Beyond experimental evidence, tannins are already exploited in application-ready products across different sectors. In the cosmeceutical field, tannin-based formulations are used in skin and hair care products, including natural hair dyes and hair-relaxing treatments, where they replace potentially harmful synthetic chemicals [9]. In parallel, standardized chestnut-derived tannin preparations are commercially available as nutraceuticals and food supplements for gastrointestinal health, exploiting their astringent, antioxidant, and barrier-supporting properties [10,11,12,13].

In this context, the valorization of AFWBs represents not only an environmentally friendly solution to reduce industrial waste and environmental burden, but also a promising scientific frontier for the identification of novel bioactive molecules with potential health-promoting properties. Such compounds—often including polyphenols, carotenoids, glucosinolates, fibers, and peptides—can exert antioxidant, anti-inflammatory, antimicrobial, and metabolic regulatory effects that benefit both human and animal health [14]. Hence, the exploitation of food industry residues aligns perfectly with the One Health vision, providing a bridge between sustainability, innovation, and preventive health, while contributing to global goals for environmental protection and resource efficiency [15].

Furthermore, many of the bioactive compounds recovered from AFWBs display biological activities that align with modern views on disease as a multifactorial process. Many diseases are now recognized as multifactorial conditions in which pathological alterations simultaneously affect multiple molecular systems. These systems interact dynamically, and restoring their balanced homeostasis may allow damaged tissues to return to a near-physiological state. Achieving such an outcome requires the modulation of several interconnected nodes within molecular networks—a strategy embodied by the multicomponent–multitarget paradigm, which is frequently observed in the biological activities of complex phytochemical mixtures [16].

Tannins are natural polyphenolic compounds found in various plants and represent one of the most emblematic examples of how AFWBs can be transformed into high-value resources. From a chemical standpoint, they are complex molecules characterized by their ability to precipitate proteins and impart an astringent taste to foods [17].

In the agro-food supply chain, tannins mainly originate from by-products and processing residues that were historically considered waste. The main sources include:

• Grape pomace and seeds from the wine industry, which account for approximately 20–25% of the processed grape weight [18,19];
• Peels and seed coats of various fruits such as lentils, carobs, and dates [20,21];
• Barks and wood residues from chestnut, oak, and other species [22,23];
• Stems and leaves derived from viticulture [19].

Tannins therefore represent a paradigmatic example of how the principles of the circular economy can be integrated into agri-food production chains [24]. According to recent analyses, the traditional linear model of the sector is showing signs of criticality, paving the way for new approaches based on the reuse and valorization of resources [25].

From a chemical perspective tannins comprise two major families—hydrolysable (gallotannins, ellagitannins) and condensed tannins (proanthocyanidins)—whose bioactivity depends on subunit composition, linkage types, and degree of polymerization [26]. Phlorotannins, produced by brown macroalgae rather than land plants, are still not consistently considered in current scholarship. Nevertheless, growing evidence over recent years points to meaningful prospects for human health, even as important uncertainties endure [27,28].

Furthermore, substantial evidence accumulated over recent decades demonstrates that each class of tannins possesses intrinsic redox-related antioxidant properties, as shown by various in vitro assays assessing radical-scavenging activity (e.g., DPPH and ABTS) and reducing power (e.g., FRAP) [29,30,31]. This strong redox-related antioxidant potential, encompassing both radical-scavenging and reducing activities, represents one of the primary—though not exclusive—mechanisms through which these compounds exert effects on animal and human health [32].

Several researchers have also attempted to maximize the yield of antioxidants obtainable from these byproducts through different approaches; indeed, a 2020 study demonstrated that acid hydrolytic treatment can effectively enhance the antioxidant activity of various agri-food byproducts, thereby providing new perspectives for their exploitation as functional additives or natural antioxidant ingredients [33].

In light of these functional properties, the recovery of tannins from agri-food by-products is increasingly being recognized as a viable strategy for their valorization within high-value applications, particularly in the nutraceutical, pharmaceutical, and breeding sectors. This integrated approach not only contributes to reducing the environmental burden associated with waste disposal but also aligns with the principles of sustainable resource management and circular bioeconomy.

Despite rapid growth of the field, the evidence base remains siloed, with human-health studies and animal performance/welfare research advancing largely in parallel and seldom integrated through common mechanistic frameworks. In this review, we bridge these silos by providing a comparative synthesis of tannin-mediated pathways—antioxidant, anti-inflammatory, antimicrobial/anti-virulence, metal-chelating and enzyme-modulating effects, protein interactions, and gut microbiota–host crosstalk (including the gut–brain axis)—across human and animal models. By mapping convergences and divergences in mechanisms, exposures, and outcomes, we derive translational implications for dose setting, formulation/standardization, and sector-specific applications.

## 2. Materials and Methods

This narrative review was conducted in three main phases: literature search, screening of abstracts and full texts, and discussion of results. To identify the relevant literature, the following databases were explored: PubMed, Scopus, Science Direct, Web of Science, and Google Scholar. The final search was completed in November 2025 and included peer reviewed international articles, online reports, and electronic books published in English.

The primary keyword used was “tannin”, combined with terms such as “agri food-waste”, “antioxidants”, “polyphenols”, “nutraceutical”, “animal”, and “health”. After the search, abstracts were reviewed to confirm their relevance to the topic. Duplicate entries were removed, and the remaining abstracts were assessed based on the inclusion criteria. Eligible studies were primarily those published within the past 10 years to ensure up-to-date scientific evidence. From the general literature screening, 7950 articles were initially identified; however, only about 0.9% met the inclusion and exclusion criteria and were deemed relevant for this review. As this is a narrative review, it was not necessary to formally register the search strategy on a specific platform.

## 3. Health Effects of Tannins

The following subsections synthesize evidence across both human and animal research. In humans, we summarize outcomes related to oxidative and inflammatory status, metabolic and vascular markers, gut barrier support, and microbiota-modulating effects, along with key considerations for safety and tolerability. In animals, we review performance and welfare outcomes in ruminants and monogastrics, covering rumen fermentation and methane emissions, nutrient utilization, antimicrobial and anti-parasitic effects, immune modulation, gut health, and growth efficiency. Throughout, we highlight dose-effect relationship, differences between condensed and hydrolysable tannins, and species-specific metabolic constraints relevant to practical applications.

### 3.1. Human Health

#### 3.1.1. Condensed Tannins on Humans

It is estimated that around 85–90% of condensed tannins escape absorption in the upper gastrointestinal tract because of their high molecular weight, polymeric structure, resistance to digestive enzymes and reach the large intestine, where microbial fermentation produces phenolic acids, valerolactones, and related metabolites [34]. Urinary flavan-3-ol metabolites from cranberry, particularly sulphated phenyl-γ-valerolactones, are biologically active at physiological concentrations and inhibit the adhesion of uropathogenic *E. coli* to bladder epithelial cells, likely representing key mediators of cranberry’s preventive effects against urinary tract infections [35]. In contrast to cranberry, green tea does not contain A-type proanthocyanidins, yet its high flavan-3-ol monomer content has been associated with reported antimicrobial effects [36].

A particularly relevant aspect of proanthocyanidin bioactivity concerns their ability to protect tissues from ischemic injury. Preclinical studies have shown that grape seed extracts (GSEs), one of the richest natural sources of proanthocyanidins [37], significantly attenuate ischemia/reperfusion damage in both hepatic and potentially cerebral tissues in rat models following oral administration [38]. This protective effect is attributed to their capacity to reduce endoplasmic reticulum stress and modulate the inflammatory response, two key processes involved in the secondary tissue injury that follows reperfusion. The combined reduction in local and systemic inflammation, together with the potent antioxidant activity of proanthocyanidins, helps limit the structural and functional damage associated with ischemic events [39]. These findings suggest that the antioxidant properties GSE may contribute also to mitigating oxidative stress in individuals with elevated cardiovascular risk, a pathological state characterized by increased systemic ROS burden [40]. GSE, rich in proanthocyanidins and gallic acid, exerts multifaceted neuroprotective effects by activating protective pathways such as Nrf2 [41] and modulating endogenous antioxidant enzymes (SOD, CAT, GSH-Px) [42], thereby enhancing cellular defense against oxidative stress, a common feature of many neurodegenerative diseases. Furthermore, GSE exhibits strong antioxidant activity by reducing reactive oxygen species, lipid peroxidation, and oxidative damage in neuronal tissue, and these combined mechanisms result in robust neuroprotection across multiple preclinical models [43].

Recent evidence highlights the multifaceted anti-obesity potential of grape seed proanthocyanidin extract (GSPE), one of the richest natural sources of condensed tannins. GSPE exerts a broad range of metabolic benefits, including significant improvements in lipid and glucose homeostasis, reductions in circulating triglycerides, leptin, and insulin, and a concomitant increase in adiponectin levels [44]. Mechanistic studies indicate that these effects stem from the modulation of key metabolic pathways, such as the activation of FXR, inhibition of JNK signaling, and downregulation of lipid-associated microRNAs (miR-33 and miR-122) [45]. GSPE also influences adipose tissue biology by limiting adipocyte hypertrophy, enhancing browning and thermogenesis in brown adipose tissue, and attenuating adipose inflammation in rats model [46]. Moreover, it favorably remodels the gut microbiota—promoting beneficial genera such as *Akkermansia* and *Bifidobacterium* in rats—and increases satiety-related hormones, thereby reducing energy [47,48]. Importantly, these findings are derived primarily from preclinical studies and therefore require validation through well-designed clinical trials. Nonetheless, collectively they support GSPE as a promising nutraceutical agent capable of counteracting obesity through complementary effects on lipid metabolism, adipose tissue function, inflammatory tone, and gut–brain axis regulation [44].

In line with these findings, a recent bio-guided investigation on *Castanea sativa* by-products demonstrated that high-molecular-weight proanthocyanidins concentrated in chestnut episperms exert potent anti-inflammatory effects by suppressing TNF-α-induced IL-8 secretion and NF-κB activation in human adenocarcinoma cells [49]. These results strengthen the view that condensed tannins from agro-industrial residues may offer targeted gastroprotective benefits.

Some authors suggest that condensed tannins purified from *Vigna angularis* seeds—predominantly procyanidins with prodelphinidins and their gallates—function as reversible, mixed-type inhibitors of tyrosinase, attenuate melanogenesis in B16 mouse melanoma cells, and exhibit strong antioxidant and DNA-protective activities [50], thereby supporting their potential application as bioactive ingredients in food, medical, and cosmetic formulations.

Additionally, Tsoupras et al. demonstrated that extracts derived from apple pomace, a byproduct rich in proanthocyanidins (condensed tannins derived from catechin and epicatechin), can be effectively used to enrich whole-grain bakery products. The incorporation of extracts obtained with food-grade solvents, in fact, significantly enhanced the bread’s antioxidant, anti-inflammatory, and antithrombotic properties on human platelets, owing to the synergistic action of simple phenolics, polar lipids, and tannins [51].

Furthermore, condensed tannins purified from *Ulmus pumila* leaves (UCTs) have been reported to exert anti-cholangiocarcinoma activity in vitro, plausibly via induction of G2/M checkpoint arrest and activation of the caspase cascade leading to apoptosis, thereby positioning UCTs as promising lead compounds for cholangiocarcinoma therapy [52].

A 2025 study have demonstrated that tannins derived from agro-industrial wood waste can be successfully incorporated into natural polymeric matrices such as chitosan to develop multifunctional hydrogels with strong antioxidant, UV-protective, and skin-regenerative properties, offering a sustainable route for the valorization of agri-food by-products in biomedical applications [53].

A further contribution to the valorization of condensed-tannin-rich matrices emerge from recent analyses of agri-food by-products, which consistently highlight apple pomace as a prominent source of procyanidins and flavan-3-ol monomers, key structural units of condensed tannins. These polyphenols, together with hydroxycinnamic acids and dihydrochalcones, constitute the dominant phenolic classes in apple residues and are strongly associated with enhanced antioxidant and antimicrobial capacities [54].

Collectively, the evidence indicates that condensed tannins—particularly proanthocyanidins from agri-food by-products—exert robust antioxidant, anti-inflammatory, metabolic, and neuroprotective effects. However, the vast majority of mechanistic insights derive from in vitro systems and animal models, where doses, exposure times, and matrix standardization are tightly controlled. Human data remain largely indirect, relying on biomarker modulation, ex vivo assays, or extrapolation from dietary intervention studies rather than on hard clinical endpoints. At present, condensed tannins should therefore be regarded primarily as proof-of-concept bioactives, with promising translational relevance but limited application-ready clinical evidence. Future progress will depend on well-designed human trials integrating pharmacokinetics, microbiota-derived metabolites, dose–response relationships, and standardized tannin characterization.

#### 3.1.2. Hydrolysable Tannins on Humans

Pomegranate peels, often regarded as food industry by-products, represent a valuable source of hydrolysable tannins, particularly ellagitannins, which exhibit remarkable antimicrobial properties. Studies have shown that aqueous extracts of pomegranate peel powder significantly reduce the population of *Escherichia coli* O157:H7 [55], demonstrating their potential as natural food preservatives. The higher ellagitannin content found in powdered peels, compared with whole peels or juice powder, correlates with their enhanced antibacterial activity [55]. Quantitative analyses identified these compounds as the major contributors to the observed antimicrobial effects [55]. Therefore, revalorizing pomegranate peel waste as a natural preservative not only supports food safety but also contributes to sustainability by reducing food waste and dependence on synthetic preservatives.

Recent investigations have evaluated a set of 25 pomegranate-derived phenolic compounds for their capacity to inhibit key targets relevant to Alzheimer disease (AD): BACE1, which is directly involved in the pathogenesis of AD, and acetylcholinesterase (AChE) and butyrylcholinesterase (BChE), which are established therapeutic targets for symptomatic treatment. Several of these compounds, particularly hydrolysable tannins such as gallagic acid, exhibited potent inhibitory activity in all three in vitro assays, highlighting their anti-AD potential [56]. Gallagic acid showed the strongest enzyme inhibition and demonstrated favorable cellular activity, molecular docking results, ADME properties, and drug-likeness profiles, supporting its potential as a bioactive neuroprotective agent [56]. In addition, recent preclinical evidence has highlighted the neuroprotective potential of ellagitannins found in *Punica granatum* peel and seeds, particularly punicalagin, confirming this matrix as a rich source of bioactive hydrolysable tannins [57]. In the study by Gonzalez-Ruiz et al. (2024), the aqueous seed extract was found to be rich in phenolic compounds [58]. In silico analyses indicated that punicalagin has strong affinity for multiple molecular targets implicated in cognitive decline, including the Epidermal Growth Factor Receptor (EGFR) and Pregnane X Receptor (PXR) [59,60], suggesting a potential multitarget mechanism relevant to neurodegeneration.

When administered in a murine model of aluminum-induced cognitive impairment, the extract produced a significant improvement in recognition memory and a tendency toward reduced glial fibrillary acidic protein (GFAP) expression in the hippocampus, indicating a possible attenuation of the astrocytic response [58]. Overall, these findings support the role of pomegranate seed ellagitannins as promising nutraceutical compounds capable of modulating key molecular pathways associated with cognitive decline and neuroinflammation.

Beyond pomegranate-derived matrices, several agri-food by-products also contain phenolic classes that are structurally or functionally connected to hydrolysable tannins, particularly through the presence of hydroxybenzoic acids, such as gallic acid, and related metabolites resulting from tannin hydrolysis. These compounds are recurrently detected in cereal brans and fruit residues, where they contribute to the overall antioxidant and antimicrobial potential of the extracts. Indeed, gallic acid and other low–molecular weight phenolics—typical degradation products of ellagitannins and gallotannins—play a central role in shaping the bioactivity of polyphenol-rich matrices, as reflected by the frequent use of gallic acid equivalents (GAE) as an analytical standard for quantifying total phenolic content in food by-products [54].

Recent metabolomic investigations on *Castanea sativa* by-products indicate that, alongside a diverse flavonoid profile, spiny burs also contain aromatic constituents attributable to condensed tannins [61]. While flavonols such as astragalin, isorhamnetin glucoside, and myricitrin dominate the leaf extracts, the burs exhibit NMR signals consistent with tannin-rich matrices previously described in chestnut residues. In this context, the authors note that tannins—well recognized for their antioxidant potential in addition to their anti-inflammatory properties—may act synergistically with flavonoids and coumarins to sustain the cytoprotective and anti-neuroinflammatory effects observed in LPS-stimulated microglia [62]. Notably, condensed tannins from *Castanea sativa* bark have demonstrated neuroprotective activity in preclinical models of oxidative and ischemic injury [63,64], highlighting their potential to prevent neuronal damage through antioxidant, cytoprotective, and anti-inflammatory mechanisms. These findings support the relevance of tannin-containing chestnut by-products as valuable nutraceutical resources with antioxidant, anti-inflammatory, and neuroprotective properties.

Hydrolysable tannins, especially ellagitannins and their derivatives, display consistent antioxidant, antimicrobial, and neuroprotective activities across in vitro, in silico, and preclinical models, including modulation of enzymatic targets relevant to neurodegenerative disorders. Nevertheless, current evidence remains predominantly preclinical, with limited validation in human intervention studies and scarce data on long-term safety, bioavailability, and metabolite-driven efficacy. While the convergence of enzyme inhibition, molecular docking, and animal cognitive outcomes supports a strong proof-of-mechanism, these findings do not yet meet the criteria for application-ready nutraceutical or therapeutic use. Bridging this gap will require controlled clinical studies focused on metabolite exposure, target engagement, and clinically meaningful cognitive or inflammatory outcomes.

### 3.2. Animal Health

#### 3.2.1. Condensed Tannins on Animals

Recent advances highlight the potential of tannin-rich agro-industrial by-products as sustainable nutraceutical tools for the control of gastrointestinal nematodes in ruminants. Evidence from both in vitro and in vivo studies demonstrates that by-products derived from hazelnuts, chestnuts, carob pods, temperate and tropical tree barks, coffee grounds, and cocoa residues possess significant anthelmintic activity, primarily linked to their content in condensed tannins and related polyphenols [65]. These compounds can interfere with multiple stages of the nematode life cycle—reducing egg hatchability, impairing larval development, and decreasing adult fecundity—thereby lowering parasite burdens and egg excretion in livestock [65].

Furthermore, condensed tannins contribute to animal health by improving protein utilization, reducing parasite burdens, and modulating gut microbial communities [66,67]. Their antioxidant and anti-inflammatory properties further support metabolic resilience, and their ability to bind toxic compounds such as ergovaline helps animals tolerate endophyte-infected forages [68]. These benefits, however, depend on maintaining intake within an optimal range, as exceeding safe tannin levels can lead to toxic effects and impair nutrient digestibility emphasizing the need for appropriate botanical sources and controlled inclusion level [66].

On the other hand, *Cryptosporidium* spp. are unicellular apicomplexan parasites that impose a major global public-health burden and cause substantial productivity losses in livestock. In the context of growing interest in plant-derived alternatives amid emerging anthelmintic and anticoccidial resistance, a 2022 study reported that the condensed tannins tested did not inhibit intracellular growth of *C. parvum* in cell culture—an apparently negative but highly informative finding [69]. This result cautions against extrapolating antiparasitic efficacy of condensed tannins observed for other parasites, underscores the scarcity of effective therapeutic options for this pathogen, and highlights the value of transparently reporting candidates with limited promise so as to refine structure–activity hypotheses and prioritize target-specific screening pipelines.

Moreover, recent evidence from poultry models indicates that dietary supplementation with GSE, a rich source of proanthocyanidins, can exert beneficial effects on lipid metabolism and oxidative status [70]. Specifically, GSE administration resulted in a significant reduction in total cholesterol and low-density lipoproteins, accompanied by a marked enhancement of endogenous antioxidant defenses, as shown by increased reduced glutathione and decreased lipid peroxidation [71]. In parallel, an improvement in the humoral immune response was observed, with higher antibody titers compared to control animals [71]. Collectively, these findings support the use of GSE as a natural antioxidant and immunomodulatory ingredient, with potentially relevant implications in a nutraceutical context [71].

#### 3.2.2. Hydrolysable Tannins on Animals

Digestion-simulated extracts from chestnut and quebracho significantly improved cell survival under oxidative stress triggered by hydrogen peroxide and dextran sodium sulfate [72]. This evidence further supports the role of tannins as protective agents against oxidative injury, which may be especially valuable in managing post-weaning diarrhea in piglets.

Recent findings also indicate that dietary supplementation with tara (*Tara spinosa* (Feuillée ex Molina) Britton & Rose) hydrolysable tannins (HT) in finishing pigs can enhance meat oxidative stability without affecting growth performance or major carcass traits. Although the fatty acid profile remained largely unchanged, meat from tara-fed animals showed lower levels of hydroperoxides, conjugated dienes, and malondialdehyde, together with a delayed onset of lipid oxidation under oxidative challenges such as catalysis and cooking [73]. These effects, likely driven by the intrinsic antioxidant and anti-inflammatory properties of hydrolysable tannins, suggest a protective role against lipid peroxidation in muscle tissues.

Beyond these findings, a broader body of evidence indicates that HT exert multiple beneficial effects on animal physiology and health. Supplementation with HT-rich agro-industrial by-products has been shown to improve feed efficiency and modulate nitrogen utilization in ruminants, while simultaneously attenuating ruminal methanogenesis through shifts in microbial populations [74]. Moreover, HTs enhance antioxidant defenses by increasing endogenous enzymatic activities and reducing markers of lipid peroxidation and inflammatory cytokines, thereby contributing to improved intestinal integrity and systemic redox balance [72]. Additional studies report antiparasitic and anticoccidial activities, alongside endothelial-protective effects linked to enhanced nitric oxide bioavailability [75]. Collectively, these data underline the multifaceted bioactivity of HT in livestock species, supporting their relevance as natural agents for improving animal health, productivity, and metabolic resilience.

#### 3.2.3. Mix Condensed and Hydrolysable Tannins on Animals

In chicken gastrointestinal smooth-muscle preparations, a HT–rich extract (from *Castanea sativa* Mill.) produced a clear, concentration-responsive reduction in contractility across intestinal segments consistent with enhanced mixing and nutrient/bile-acid absorption and firmer stool consistency. On the other hand, the condensed–dominant tannin extract (from *Schinopsis balansae* Engl.) showed a less favorable, segment-dependent modulation, suggesting that the predominance of condensed tannins, with stronger protein-binding and astringent properties, may differentially interact with smooth-muscle receptors or ion channels, resulting in a non-uniform regulation of intestinal motility along the gastrointestinal tract. Nonetheless, the authors note potential complementarity that warrants testing combined regimens in poultry [76].

Recent meta-analytical evidence further supports the relevance of botanical sources of tannins in shaping productive and qualitative outcomes in sheep. While dietary tannins generally do not alter dry matter intake, they can enhance growth performance, antioxidant capacity, and carcass traits. Notably, improvements in feed efficiency and antioxidant status are most consistently associated with condensed tannins naturally present in plant ingredients. Reductions in lipid oxidation in plasma and meat are especially evident when condensed and hydrolysable tannins are provided in combination, with *Vitis vinifera*, *Hedysarium coronarium*, *Sorghum bicolor*, *Pisum sativum*, and *Pistacia vera* emerging as the most effective botanical sources [77].

Complementary findings emerge from studies in ruminants, where tannin-rich botanical by-products can be integrated with other functional feed components. For example, supplementing beef cattle diets with green tea by-product tannins in combination with biochar has been shown to sustain feed intake while improving growth performance and nutrient efficiency [78]. At moderate inclusion levels, this strategy also leads to meaningful reductions in methane emissions without impairing digestibility, highlighting the potential of tannin-containing residues to simultaneously support animal performance and reduce environmental impact [78].

## 4. Discussion

The present review highlights how tannin-rich agri-food by-products represent a versatile and underexploited resource capable of delivering significant benefits across both human and animal health domains. Although tannins have traditionally been regarded primarily as astringent or antinutritional factors, recent evidence demonstrates a far more complex biochemical role that spans antioxidant, anti-inflammatory, antimicrobial, antiparasitic, metabolic, and neuroprotective pathways. These biological effects emerge from the structural diversity of tannins—namely condensed, hydrolysable, and mixed tannin matrices—which modulate distinct yet often convergent molecular targets. The diversity of activities, summarized in Table 1, underscores this multifunctionality and provides a conceptual framework for interpreting sector-specific applications.

Across human health studies, condensed tannins—particularly proanthocyanidins from grape seed, apple pomace, and legume or tree-derived sources—consistently demonstrate potent antioxidant and cytoprotective effects. These activities extend beyond simple radical scavenging and involve the modulation of redox-sensitive transcription factors, such as Nrf2, and the attenuation of inflammatory signaling. Notably, several lines of evidence link condensed tannins to metabolic benefits, including improved lipid handling, reduced adipose inflammation, and modulation of gut microbiota composition toward health-promoting genera. HT, especially ellagitannins and their metabolites, show complementary neuroprotective and antimicrobial actions, including inhibition of key target implicated in the onset and therapy of AD and reduction in clinically relevant bacterial pathogens. Together, these findings support the view that tannins exert systems-level effects relevant to chronic disease prevention and gut–brain axis modulation—although robust, well-controlled clinical studies remain limited.

**Figure 1:**
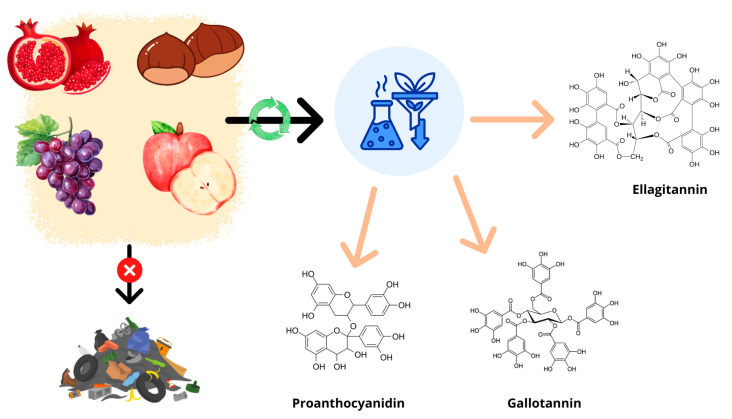
Agri-food waste and by-products, often destined for landfill disposal (red sign arrow), can be subjected to extraction processes to recover tannins (green sign arrow), thereby transforming food waste into a resource with high bioactive value.

In the context of animal health, condensed and HT display similarly broad functional roles but with added relevance to livestock production and sustainability. Consistent evidence supports their capacity to reduce gastrointestinal nematode burdens, improve immune status, and enhance antioxidant defences. In ruminants, tannins can modulate rumen fermentation patterns, improve nitrogen utilization and reduce methane emissions—an outcome aligned with global sustainability goals. The dual capacity to enhance animal performance while mitigating environmental impacts makes tannin-containing by-products particularly attractive for circular-agriculture strategies (Figure 1). However, dose dependency and species-specific metabolic constraints must be carefully considered, as excessive tannin intake may impair nutrient digestibility or attenuate beneficial effects.

Importantly, several studies reveal synergistic effects when condensed and hydrolysable tannins coexist within the same botanical matrix or feeding strategy. Mixed tannin systems appear to provide broader antioxidant protection, stronger anti-parasitic activity, and more consistent improvements in meat quality traits compared with single-class tannins. This observation reinforces the need for a nuanced approach to tannin standardization in functional feeds and nutraceuticals, emphasizing the role of compositional profiling over reliance on generic measures such as total tannin content.

The available evidence summarized herein predominantly derives from in vitro and animal studies, whereas well-controlled clinical investigations remain scarce. Consequently, many of the reported effects should be interpreted primarily as proof-of-concept rather than as application-ready outcomes, particularly given the marked heterogeneity in study design, botanical sources, tannin profiles, and administered doses.

From a mechanistic standpoint, the antioxidant activity of tannins represents a proximal event that modulates multiple downstream biological pathways. By limiting excessive ROS accumulation, tannins may reduce the activation of redox-sensitive transcription factors such as NF-κB and AP-1, thereby attenuating the expression of pro-inflammatory cytokines and enzymes. In parallel, preservation of redox homeostasis supports mitochondrial function, may limit lipid peroxidation, and helps prevent oxidative damage to neuronal membranes and proteins, providing a mechanistic basis for the observed neuroprotective effects. Furthermore, activation of endogenous antioxidant pathways, including Nrf2-mediated signaling, may contribute to long-term cytoprotective and metabolic adaptations, linking antioxidant activity to broader anti-inflammatory, neuroprotective, and metabolic outcomes.

Despite these promising findings, several knowledge gaps remain. First, the mechanistic underpinnings of tannin bioactivity, particularly in complex biological matrices, are far from being fully elucidated. In both humans and animals, the interplay between tannins, the gut microbiota, and downstream metabolites demands deeper investigation, especially considering the emerging relevance of the gut–brain and gut–immune axes. Second, there is a need for harmonized methodologies in tannin extraction, characterization, and quantification to ensure reproducibility and cross-study comparability. Third, the translation of in vitro and preclinical findings into effective, safe, and standardized applications for human health requires larger, well-designed clinical trials.

## 5. Conclusions and Future Perspectives

Overall, the collective body of evidence reviewed here demonstrates that tannin-rich agri-food by-products hold considerable promise as sustainable, multifunctional bioactive antioxidant resources. Their integration into nutraceutical, food, pharmaceutical, and livestock sectors aligns well with the One Health and circular bioeconomy paradigms, offering a strategic opportunity to convert waste streams into high-value functional ingredients. Future research should prioritize well-defined and feasible strategies to improve the translational value of tannin-based interventions. In particular, greater emphasis should be placed on the chemical standardization and compositional profiling of tannin-rich matrices, alongside dose–response and bioavailability studies integrating gut microbiota–derived metabolites. In parallel, mechanistic investigations linking redox modulation to specific inflammatory, metabolic, and neuroprotective biomarkers would strengthen causal interpretation. Finally, clinical studies should be designed around clearly defined endpoints and target populations, rather than exploratory supplementation trials, to facilitate the progression from proof-of-concept to application-ready evidence. Continued interdisciplinary efforts will be essential to optimize extraction processes, clarify biological mechanisms, establish effective dose ranges, and ensure the safe and sustainable use of tannins across human and animal applications.

## Figures and Tables

**Table 1:** Overview of tannin types, main activities, and corresponding references.

Tannins “Type”	Main Activities	References
Condensed tannins (humans)	Poor absorbability, formation of microbial metabolites; anti-ischemic effect; neuroprotection via Nrf2; antioxidant activity; anti-obesity effects (FXR, JNK, miRNA modulation, adipose browning); tyrosinase inhibition; enhancement of bread antioxidant/anti-inflammatory properties; anticancer (cholangiocarcinoma) activity; hydrogel bioactivity; antimicrobial & antioxidant potential in apple pomace; anti-gastritis effects via inhibition of TNF-α–induced.	[26,27,28,29,30,31,32,33,34,35,36,37,38,39,40,41,42,43,44,45,46]
Hydrolysable tannins (humans)	Antimicrobial activity vs. *E. coli* O157:H7; cholinesterase & BACE1 inhibition (anti-AD); neuroprotection (punicalagin, EGFR/PXR interactions, anti-GFAP); antioxidant & antimicrobial contributions of gallic acid; anti-inflammatory synergy in chestnut by-products.	[47,48,49,50,51,52,53,54,55,56]
Condensed tannins (animals)	Anthelmintic activity vs. gastrointestinal nematodes; improved protein utilization; modulation of gut microbiota; antioxidant/anti-inflammatory support; ergovaline-binding tolerance; species- and dose-dependent effects; no activity against Cryptosporidium; improved lipid metabolism, immune response, antioxidant markers in poultry.	[57,58,59,60,61,62,63]
Hydrolysable tannins (animals)	Cytoprotection under oxidative stress (H_2_O_2_, DSS); improved meat oxidative stability (pigs); enhanced rumen fermentation profile, reduced methane; improved antioxidant enzyme activity & reduced inflammation; anticoccidial, antiparasitic, vasoprotective effects.	[64,65,66,67]
Mixed condensed & hydrolysable tannins (animals)	Modulation of intestinal contractility (chicken); improved growth and antioxidant capacity (sheep); reduced lipid oxidation when mixed tannins are provided; reduced methane emissions and improved feed efficiency when combined with biochar.	[68,69,70]

## Data Availability

No new data were created or analyzed in this study. Data sharing is not applicable to this article.

## Abbreviations

The following abbreviations are used in this manuscript:

ABTS	2,2′-Azino-bis(3-ethylbenzothiazoline-6-sulfonic acid)
AChE	Acetylcholinesterase
AFWBs	Agri-Food Waste and By-Products
BACE1	β-site Amyloid Precursor Protein–Cleaving Enzyme 1
BChE	Butyrylcholinesterase
CAT	Catalase
DPPH	2,2-Diphenyl-1-picrylhydrazyl
DSS	Dextran Sodium Sulfate
EGFR	Epidermal Growth Factor Receptor
FRAP	Ferric-Reducing Antioxidant Power
GAE	Gallic Acid Equivalents
GFAP	Glial Fibrillary Acidic Protein
GSE	Grape Seed Extract
GSPE	Grape Seed Proanthocyanidin Extract
GSH-Px	Glutathione Peroxidase
H_2_O_2_	Hydrogen Peroxide
HT/HTs	Hydrolysable Tannins/Hydrolysable Tannins (plural)
IL-8	Interleukin-8
miRNA	microRNA
NF-κB	Nuclear Factor kappa-light-chain-enhancer of activated B cells
NMR	Nuclear Magnetic Resonance
Nrf2	Nuclear Factor Erythroid 2–Related Factor 2
PXR	Pregnane X Receptor
ROS	Reactive Oxygen Species
SOD	Superoxide Dismutase
UCTs	Ulmus pumila Condensed Tannins
